# Dr. William Harland Peake

**Published:** 1910-06-18

**Authors:** 


We have to announce the death of Dr. William Harland Peake, at the age of forty. He studied at Guy's Hospital, and graduated M.D. of Durham in 1897, having taken his diploma and his B.S. in 1895. Dr. Peake had held appointments at the Royal Westminster Ophthalmic Hospital and the Hospital for Women and Children, Soho Square.

